# Summary

**Published:** 1881-10

**Authors:** 


### Summary.

### VACCINATING FOR ANTHRAX ON A LARGE SCALE AND SUCCESSFULLY. By PASTEUR.

In April last, the Agricultural Society of Melun proposed to me, through its president, the Baron de la Rochette, to settle, by decisive experiments, the results announced by me in the Academy. I consented, and April 28 the following agreement was made:

1. The Agricultural Society of Melun shall place sixty sheep at the disposal of M. Pasteur.

2. Ten of these sheep shall receive no treatment.

3. Twenty-five of these sheep shall be vaccinated twice, with an interval of twelve days, two unequally attenuated viruses being used for that purpose.

4. These twenty-five sheep, as well as the twenty-five remaining, shall be inoculated with very virulent anthrax, after another interval of twelve or fifteen days.

The twenty-five unvaccinated sheep shall all die; the twenty-five vaccinated sheep shall resist, and shall be compared ultimately with the ten sheep kept in reservation, in order to show that the vaccinations do not prevent the animals from returning to a normal state.

5. After this general inoculation of very virulent virus to the two sets of twenty-five sheep, vaccinated and not vaccinated, these fifty sheep shall remain together in the stables; one of the series being distinguished by punching the ear of the twenty-five vaccinated sheep.

6. All those sheep which anthrax will kill shall be separately buried in an inclosed space.

7. In May, 1882, twenty-five fresh sheep, never used before in experimenting, shall graze in this space, in order to show that fresh sheep will spontaneously contract anthrax from the germs of the disease which earth-worms shall have brought from the cadavers to the surface of the ground.

8. Twenty-five more fresh sheep shall graze around this inclosed space, a few meters off, where no cadaver has ever been buried, in order to show that none of them shall die from anthrax.

Addition to the preceding convention programme. — The President of the Agricultural Society of Melun having manifested a desire to have these experiments include cows, I gave my consent, remarking, however, that until now, the experimental vaccination of cows had not been carried to such an extent as that of sheep, and that consequently the case might be that such experiments might not be so encouraging as those on sheep. In any case, I expressed my gratitude to that society for placing ten cows at my disposal, in order that six might be vaccinated and four left alone, and that after this vaccination the ten cows should be inoculated at the same time as the fifty sheep with most virulent virus. I affirmed, on one hand, that the six cows previously vaccinated would not be sick, while the four unvaccinated cows should perish partly or in a whole, or at least would be very sick.

I own such a programme showed a boldness in prophesying that only a brilliant success could warrant. The experiments were begun May 5, in the commune of Pouilly-le-Fort, near Melun, on a farm belonging to M. Rossignol.

In order to satisfy the wishes of the Agricultural Society which started the experiments, it was agreed to replace two sheep by two goats, and, as no condition of age or race had been proposed, it happened that the fifty-eight sheep differed in age, sex and race. Among the ten beeves were eight cows, an ox and a bull.

On May 5, 1881, were inoculated by hypodermic injection twenty-four sheep, a goat, and six cows, five drops of an attenuated anthrax virus being injected into each animal. May 17 we re-inoculated these twenty-four sheep, the goat, and the six cows with another attenuated virus more powerful than the first.

May 31, we proceeded to vaccinate with the most virulent virus, in order to test the efficacy of former vaccinations. In order to do that, we inoculated on one hand the thirty-one beasts just spoken of, on the other, twenty five sheep, a goat, and four cows—none of the latter having been previously vaccinated.

The most virulent virus, used May 31, was one regenerated from the germinal corpuscles of the anthrax bacteria, which I had kept in my laboratory ever since March 21, 1877. In order to insure more comparative experiments, a vaccinated animal and one not vaccinated were alternately inoculated. June 2, the results were wonderful. The twenty-four sheep and the goat which had been vaccinated, together with the six cows, were all in good health ; on the contrary, twenty-one sheep and the goat, which had not been vaccinated, had already died of anthrax. The three other sheep of this series died the same day.

The non-vaccinated cows were not dead, being less liable to die from the disease than sheep ; but all had large swellings about the point of inoculation. Their temperature went up three degrees. The vaccinated cows had no rise in temperature, no tumor, and no inconvenience; and this shows the vaccination of cows to have been as successful as that of sheep.

ST. ANN ASYLUM. Service of PROF. BALL.

*Syphilitic Insanity.*—The influence of diatheses on the evolution of mental affections is now an uncontested fact. Rheumatism and gout both give rise to real insanities, which we studied in our preceding lessons under the names of rheumatic and gouty insanity. Syphilis, as the other diatheses, can also originate insanity. You know that syphilis, after it has attacked the skin and the mucous membranes, the bones, the muscles and the cellular tissue, finally takes to the viscera, and causes well-known lesions in the various parts of the nervous system. Presently, cerebral syphilis is no longer contested, and it was masterly investigated by Prof. Fournier. Anteriorly to this writer, Dagonet had sustained and proved the existence of syphilitic insanity, of which he had met a few cases at St. Ann Asylum. In Germany, syphilitic psychoses have been investigated. We shall in turn resume our actual knowledge of that affection.

And first, to what lesion shall we refer this special form of insanity, caused by syphilis? To cerebral lesions? They are so numerous, indeed, that one is only embarrassed by the choice. To an alteration of the blood? or to an unknown cause? This point has not yet been settled.

In a syphilitic patient, at what time in the course of the disease, at what age of the diathesis, says Fournier, do the cerebral manifestations appear? . . . These may come sooner or later. Although in some cases they are manifested a few months only after the original chancre, in others they may not appear till twenty or thirty years later. The medium is about the tenth year, but the exceptions are numerous.

Why should syphilis attack the brain? Here let us note that it most generally occurs in persons whose general health has much suffered, and who present that state called venereal cachexia, or syphilitic chlorosis. Beside the constitution, there may be exciting causes; for instance, the various venereal excitements, excesses of coition following long continence, which have so great an influence over mental manifestations, which are thereby perverted and disturbed in people predisposed. Add to this a powerful and preponderant cause, alcoholic excesses. Take into consideration fatigues and excesses of intellectual work, which have made the brain a *locus minoris resistantiæ;* this is an influence easily noticed among writers, lawyers, politicians, who are much more often attacked by syphilis than the lower classes, among which it is rather rare. Finally, a last cause, which no one could deny, traumatisms, strokes, and shocks to the head.

This being admitted, let us make another query before forming a judgment. Which kind of syphilis most likely originates cerebral lesions? You are aware, gentlemen, that there are benign, common and malignant syphilis; these are specially manifested by the rapidity of their course, and cutaneous lesion. Cerebral lesions are mostly excited by syphilis of medium intensity; yet we should fear the mild forms, sometimes very insidious, and not forget that there are abortive forms in which secondary manifestations may not appear. Let us now come to the symptoms of syphilitic insanity. We will overlook the other manifestations of cerebral lesions: paralyses, aphasia, amnesia, etc., which sometimes precede or accompany the disease under consideration. The first intellectual accident may suddenly appear. According to Fournier, insanity is often introduced by a notable failing of the intellect; then comes the syphilitic delirium, which may present itself under three forms :

1. Syphilitic dementia.
2. Syphilitic mania or insanity.
3. General syphilitic pseudo-paralysis (Fournier).

1. *Syphilitic dementia.*—The sufferer from syphilis, after a certain period, changes his moods. Some disturbance of the intellect, and peculiarities of his behavior are noticed. One who was before polite, active, bright, witty, becomes by degrees morose, impolite, dull, numb, reticent, speechless ; intellectual labor is difficult, work painful, tiresome, even unbearable, in the discharge of the common duties of life. Fournier considers loss of memory a remarkable phenomenon. For my part, gentlemen, that is a trivial phenomenon; for of the various faculties of the brain, memory is the most delicate and fallible. The judgment, on the contrary, often remains right. Syphilitic amnesia may be slow and continuous, or arise suddenly. The loss of memory is sometimes almost complete, and the patient, as do some old men, may have none but youthful recollections left, recent events being forgotten ; some patients even forgetting their children. Others have forgotten the simplest rules in arithmetic ; others a foreign language ; others at last have lost their notion of time and distance. Add to this an absolute incoherence of ideas and actions. The patient now reaches a complete dementia ; being mean, filthy, disgusting and coarse. Besides a defective moral sense, and peculiar habits, the patient is so irritable as to get mad at anything, though his anger is short. To resume, this is a premature senility of mind, differing from common old age in this, that it can be cured.

2. *Syphilitic mania or insanity.*—Syphilitic delirium, as well as dementia, may appear with or without prodroma. In some there is a stage of excitement, characterized by a feverish activity of the individual, who is restless, speaks much, and forms a quantity of projects. This soon passes into frenzy and insanity ; this frenzy appears under various forms. Sometimes it is a properly syphilitic mania, in which the patient, suffering from a true muscular delirium, breaks everything which comes in his way; his words are incoherent, there is complete insomnia, as in the excitement stage of general paralysis; this might lead into some error of diagnosis, but these acute symptoms are more marked in true mania, while there is no other differential symptom. Besides that form, lypemania, with a tendency to suicide, may appear in some subjects; 2, then hypochondria in all its forms, syphilophobia especially; 3, the delirium of persecution, though vague and confused; 4, last, and this is important, there is sometimes an impulsive delirium, especially in syphilitic epileptics. A delirium of hallucination has also been described.

3. *General syphilitic paralysis.*—You are aware, gentlemen, that some authors believe syphilis the chief cause of general paralysis, while others think it has no influence in this respect. The truth lies between the two, and Fournier admits the existence of a peculiar form of syphilis of the brain which gives rise to a general pseudo-paralysis. This is manifested by an incoherence in the intellect, both in words and writings. An ambitious frenzy is rare; patients being generally dissatisfied with themselves, and they sometimes have excesses of low melancholy, as in general paralysis. Their speech is often impeded, and they stammer from paralysis, not from a muscular contraction, as is the case in general paralysis. Besides, in syphilis there is no tremulousness of the fibrillæ of the tongue, of the upper lip, nor of the facial muscles, but there is sometimes partial paralysis, especially in one eye.

A true diagnosis can be made through complications and concomitant symptoms mainly; as cephalalgia, alopecia, gummata; but especially through partial paralyses, an important indication of syphilis of the brain.

" Strabismus, it has been said, suggests syphilis." Finally, an exhaustive and severe inquest should be made on the body of the patient, his history looked into, and not only should his family be questioned, but especially his comrades.

Whenever the patient comes under timely treatment, the prognosis is good. Hence it is necessary to act promptly and energetically. At first the mild treatment: potassium iodide, 5

grains at first, and inunctions of mercury, without any fear of any bad results from such a course (Fournier). Alternate medication is better tolerated. In case mercury could not be borne, chloride of gold may be beneficial. Finally, one must not forget that there are bad cases in which, in spite of any treatment, syphilis results in death.

MANAGEMENT OF LABOR, WITH THE PATIENT IN THE ENGLISH POSITION. By Dr. Rendu.

Labor on the side, also called the English position, because women are delivered in that way in England, is also the manner of delivery in most of the lying-in hospitals of Switzerland and Germany, but only in primiparæ. Multiparæ are delivered on their back, in France, because the elasticity of the external genital organs protects them more against lacerations of the perineum. In fact, the chief advantage of that method is to render much less frequent lacerations of the perineum in primiparæ. One can thus survey very well its progressive distension, and that of the vulva, and consequently one can appreciate better the support which the parts require ; beside, the accoucheur is in a better position to give a more efficient support.

It is no exaggeration when I state that in France nearly one-half of primiparæ suffer more or less laceration of the perineum. In Vienna's Lying-In Hospital, however, where more than 9,000 women are annually delivered, the proportion of such lacerations, in primiparæ, is six per cent.; it is ten per cent., if incisions with a protected bistoury are considered as lacerations.

But, to obtain such results, I must say that it is not sufficient at the time of birth that the mother should lie on her side, and that the perineum be more or less supported ; one needs some practice beside. Thanks to the courtesy of Prof. C. Braun, and his two assistants, Drs. Pawlik and Welponner, I spent three nights in Vienna's Lying-in Hospital, where I performed twenty-one deliveries on the side, under the direction of the hospital midwives, who are very experienced in the matter.

Here is briefly the *modus operandi:* As soon as the head appears at the vulva, the woman is made to lie on her left side, her right leg being raised and held by an assistant. The accoucheur, standing on the right of the parturient woman, passes his left hand between the woman's thighs, carrying it forward and applying it against the child's head. He supports the perineum with his right hand ; but the resistance thus afforded must not be a passive one. He must, on the contrary, during each labor pain, press energetically over the sacro-coccygeal region, and pull as much integument as he can over the child's head. Meanwhile, his left hand steadies the head at the vulva and prevents its coming out under the *influence of uterine contractions.* In the interval between the pains, the head goes back, soon to return again. This forced alternate motion which the head undergoes has for its result the gradual distension and a greater elasticity of the vulva. At last, the head comes out and extension takes place. One must carefully prevent this expulsion from taking place during a uterine contraction, and let the head come out when the pain is nearly over. The perineum must be supported to the end, for the passage of the shoulders is ordinarily more dangerous to the fourchette than that of the head itself. This is the manner, writes Dr. Rendu to the *Lyon Médical*, in which I deliver primiparæ at the clinique of the Charité of Lyon, and Prof. Bouchacourt insists on that method in his lectures before the medical classes.—*Le Médecin Praticien*, March 12.

For 5,100 medical students in 1880 in Paris, there were only two chairs of obstetrics, one of theory and one of practice. In London and Manchester there were eighteen for 2,421 students ; in Scotland, six chairs, and 1,566 students ; in Austria, six chairs, and 1,505 students ; in Belgium, four chairs, and 238 students ; in Switzerland, three chairs, and 404 students ; in Germany, twenty-one chairs, and 4,405 students ; in Italy, seventeen chairs, and 3,455 students. While there is thus a chair of obstetrics to 187 students in the rest of Europe, there is but one to 2,550 students in Paris, and only one clinical service for 5,100 students.

